# Precise regulating the specific oxygen consumption rate to strengthen the CoQ_10_ biosynthesis by *Rhodobater sphaeroides*

**DOI:** 10.1186/s40643-024-00813-0

**Published:** 2024-11-05

**Authors:** Bo Li, Yan Ge, Jianguang Liang, Zhichun Zhu, Biqin Chen, Dan Li, Yingping Zhuang, Zejian Wang

**Affiliations:** 1 State Key Laboratory of Bioreactor Engineering, East China University of Science and Technology, 130 Meilong Rd, P.O. box 329#, Shanghai, 200237 China; 2 College of Pharmaceutical and Life Sciences, Changzhou University, Changzhou, 213164 China; 3 Inner Mongolia Kingdomway Pharmaceutical Company, Hohhot, 010000 China

**Keywords:** Coenzyme Q_10_, Specific oxygen consumption rate, *Rhodobater sphaeroides*, Dielectric spectroscopy, Morphology

## Abstract

Coenzyme Q_10_ (CoQ_10_) is the most consumed dietary supplement and mainly biosynthesized by aerobic fermentation of *Rhodobacter sphaeroides* (*R. sphaeroides*). Oxygen supply was identified as a bottleneck for improving CoQ_10_ yield in *R. sphaeroides*. In this study, a precise regulation strategy based on dielectric spectroscopy (DS) was applied to further improve CoQ_10_ biosynthesis by *R. sphaeroide*. First, a quantitative response model among viable cells, cell morphology, and oxygen uptake rate (OUR) was established. DS could be used to detect viable *R. sphaeroides* cells, and the relationship among cell morphology, CoQ_10_ biosynthesis, and OUR was found to be significant. Based on this model, the online specific oxygen consumption rate (Q_O2_) control strategy was successfully applied to the CoQ_10_ fermentation process. Q_O2_ controlled at 0.07 ± 0.01 × 10^− 7^mmol/cell/h was most favorable for CoQ_10_ biosynthesis, resulting in a 28.3% increase in CoQ_10_ production. Based on the multi-parameters analysis and online Q_O2_ control, a precise online nutrient feeding strategy was established using conductivity detected by DS. CoQ_10_ production was improved by 35%, reaching 3384 mg/L in 50 L bioreactors. This online control strategy would be effectively applied for improving industrial CoQ_10_ production, and the precise fermentation control strategy could also be applied to other fermentation process.

## Introduction

CoQ_10_, also known as 2,3-dimethoxy-5-methyl-6-polyprenyl-1,4-benzo-quinone, is currently the third largest nutritional supplement, following fish oil and various vitamins (Arenas-Jal et al. 2020). CoQ_10_ is mainly biosynthesized by *R. sphaeroides*, which has the highest yield in native hosts, stands out as the optimal choice for the industrial scale production (Lee et al. 2017) (Shukla and Dubey 2018; Yuan et al. 2012). Since the biosynthesis of CoQ_10_ by *R. sphaeroides* is an aerobic fermentation, oxygen plays a critical role. Recent research has reported that through controlling the oxygen uptake rate (OUR) by adjusting the oxygen supply level, CoQ_10_ production was improved 15.5% (Z.-J. Wang et al. 2020). Although, OUR is a useful and practical online regulating measure (Zhang et al. 2004), specific oxygen uptake rate (Q_O2_) can truly reflect the oxygen uptake rate of the cell (Guo et al. 2016). Through Q_O2_ controlling, vitamin B_12_ production was improved 14.7% in *Pseudomonas denitrificans (P. denitrificans)*. Moreover, Q_O2_ also influenced the *P. denitrificans* morphology (Z.-J. Wang, Shi, et al., 2016). Cell morphology have huge impact on production titer, and its change serves as a signal for microbial to switch from growth to biosynthesis (Cui et al. 2016; Ibrahim 2015). In the process of PHA fermentation, PHA production is accompanied by cell expansion (Li et al. 2014; Shantini et al. 2015). In the CoQ_10_ fermentation, Yoshida found that the oxygen supply level affects cell morphology, thereby affecting the CoQ_10_ biosynthesis (Yoshida et al. 1998). Even though oxygen has significant effect on CoQ_10_ biosynthesis and cell morphology, the relationship between Q_O2_, CoQ_10_ biosynthesis, and cell morphology has hardly been reported.

To accurately evaluate the effects of Q_O2_ on cell morphology changes and CoQ_10_ biosynthesis, it is critical to accurately monitor the number of viable cells. Traditional approaches for measuring biomass include dry cell weight (DCW), optical density (OD), and colony forming units (CFU) (Xiong et al. 2008). However, neither OD nor DCW provides an accurate measure of viable cells (Lawrence and Maier 1977; Xiong et al. 2008).While CFU can measure viable cells, the results always lag behind and exhibit larger errors (with relative standard deviations around 10–30%), which cannot meet the requirement for precise online monitoring and fermentation control (Balestra and Misaghi 1997; Mizuochi and Nelson 2016). To more accurately determine the viable cells count in fermentation, DS is now widely used in industrial fermentations (Zitzmann et al. 2018). DS can accurately detect viable cells on line and in real time through capacitance, as dead cells have no contribution to the capacitance signal (Kiviharju et al. 2008). It has been widely used to track the concentration of several types of cells, including bacteria (Carvell and Dowd 2006), yeast (Kedia et al. 2013), and mammalian cells (Blewett et al. 2013; Rowe et al. 2003). For instance, during the fermentation process of recombinant proteins in *Drosophila melanogaster* S2 Cells, using DS to detect viable cells online and real-time led to a 5-fold increase in protein production (Zitzmann et al. 2018). However, the application of DS in the fermentation of *R. sphaeroides* remains scarce.

In this study, we accurately assessed the impact of Q_O2_ on CoQ_10_ biosynthesis and cell morphology by using DS to monitor viable cells during *R. sphaeroides* fermentation for the first time. Furthermore, a multi parameters quantitative response model was established, which included the viable cells detected, the relationship of *R. sphaeroides* morphology changes with OUR and CoQ_10_ biosynthesis discovered, and the Q_O2_ level optimized. Based on the model, and combined with the online conductivity detected by DS, the fermentation process of CoQ_10_ was optimized, and the yield of CoQ_10_ was improved obviously.

## Materials and methods

## Microorganism and nutrient medium

The strain used in this study was *R. sphaeroides*, which was donated by Inner Mongolia kingdomway Pharmaceutical Co., Ltd.

The seed medium contained (in g/L) ammonium sulfate 3.0, aginomoto 0.70, corn steep powder 0.70, glucose 15, yeast extract 2.0, NaCl 1.0, K_2_HPO_4_ 0.7, KH_2_PO_4_ 0.70, NH_4_Cl 1.00, MgSO_4_ 3.60, ferrous sulfate 0.17, and pH 7.0. The fermentation medium contained (g/L) ammonium sulfate 3.6, aginomoto 3.6, corn steep powder 3.6, KH_2_PO_4_ 2, MgSO_4_ 5.6, ferrous sulfate 1.2, copper sulfate 2.5, yeast cream 0.6, Calcium carbonate 2.5 and pH 6.7. The feed medium contained (in g/L) glucose 600 g.

The nutrient composition contained (in g/L) NH_4_SO_4_ 60 g, aginomoto 80 g, corn syrup dry powder 400 g, MgSO_4_ 100 g, FeSO_4_ 20, NaSO_4_ 10 g, yeast extract 10 g, dissolved in 3 L deionized water.

### Culture method of *R. sphaeroides*

Single colonies in the plate culture were inoculated into a seed flask with a working volume of 100mL and grown at 260 rpm and 32 °C for 28 h. The cultured seed liquid (1.5 L) was then inoculated into a 50 L bioreactor containing 30 L of fermentation broth, equipped with devices to monitor online parameters. The aeration rate was set at 0.8 vvm, the pressure at 0.05 Mpa, and the agitation speed at 550 rpm, with the culture temperature controlled at 32 °C. During the fermentation process, the residual glucose concentration was maintained at 5.0 ± 0.1 g/L through continuous feeding of glucose solution, with the feed medium added at 20 h. The pH was controlled at 6.7 using ammonium hydroxide during the fermentation.

### Detection of bacteria concentration

The optical density of the fermentation broth was measured using an ultraviolet spectrophotometer at a wavelength of 700 nm (OD_700_). The broth was diluted to an appropriate multiple, and the measured absorbance was multiplied by the dilution factor to obtain the optical density. The capacitance was measured using the DS, selected frequency was 680 kHz (Z.-J. Wang, Shi, et al., 2016).

### Standard curve of *R. sphaeroides* reference materials

*R. sphaeroides* reference materials of 0, 0.325, 0.65, 0.975, 1.3, 1.625, 1.95, 2.275, 2.6 g/L were configured as 50 ml of standard solutions, and their capacitance was measured to establish a standard curve between DCW and capacitance. Subsequently, the standard solution containing 2.6 g/L reference materials was diluted to OD values of 0.2, 0.4, 0.6, and 0.8, respectively, using sterile water as a control. Then, the standard solution of different OD was diluted to an appropriate suitable multiple to coat the plate. After 48 h of culture, plates containing 30–300 CFU of typical *R. sphaeroides* colonies were selected and calculated according to Eq. 1. The relationship between OD and CFU was then established. In the form, N is the concentration of viable cells in the sample suspension; Ci is the typical number of colonies on a plate with a certain dilution; m is the number of plates with a certain dilution; v is the inoculation volume; d is the dilution factor.

1 $$\:\:N=\frac{\sum\:_{i=1}^{m}{C}_{i}}{mvd}$$

### Microbial morphology analysis

To determine the fermentation processes bacterial sizes, Image-Pro Plus software (Media Cybernetics, Bethesda, MA) was used to analyze image (Z.-J. Wang, Shi, et al., 2016). First, light microscope images of bacteria acquired by a CCD camera (cells are magnified 100 times) were handled with best-fit equalization and contrast enhancement. Then, the effects of edge enhancement, noise cancellation and smoothing were realized by binary processing. Finally, the counted dark areas are the cells projected areas under the microscope. In parallel, the uninteresting objects must be hidden. In this investigation, three repetitions were conducted at every time point, and the number of bacteria counted not less than 50.

### Determination of the optimal QO2 level

The Q_O2_ of the fermentation process was calculated by the OUR and capacitance, which were collected online by the mass spectrometer and DS (Liang and Yuan 2006). Different Q_O2_ levels were controlled to investigate their influence on CoQ_10_ biosynthesis (Z.-J. Wang, Shi, et al., 2016) .

### Nutrients feeding strategy directed by On-line conductivity

In the 50 L bioreactor, the conductivity was controlled by feeding the nutrients in the late stage of the fermentation process (Z.-J. Wang et al. 2016b). The effects of nutrient feeding strategies were investigated. Control group without added nutrients. A and B groups started feeding nutrients at 50 h and 60 h respectively, and the conductivity was maintained at a different stable level by controlling the feeding rate.

### CoQ10 concentration detection

2.5 ml of fermentation broth was acidified by adding 1 drop of 6 mol/L hydrochloric acid in a 50 ml centrifuge tube. 5 ml of acetone and 0.5 ml of hydrogen peroxide were then added. Afterward, 99.99% alcohol was added to dilute to 25 ml. The final step was ultrasound for 45 min. The treatment solution was used for product detection with HPLC. Detection conditions: C18 chromatography column, mobile phases is methanol and ethanol, flow rate of 1.1 ml/min, column temperature of 37℃, detection wavelength of 270 nm (Z.-J. Wang et al. 2020).

### Statistical analysis

The t-test was used to evaluate statistical differences, and samples with *P* < 0.05 were considered significant. Data were analyzed using Origin Pro8.5 software.

## Result and discussion

### Building model for detecting viable *R. sphaeroides* cell by DS

**Fig. 1:**
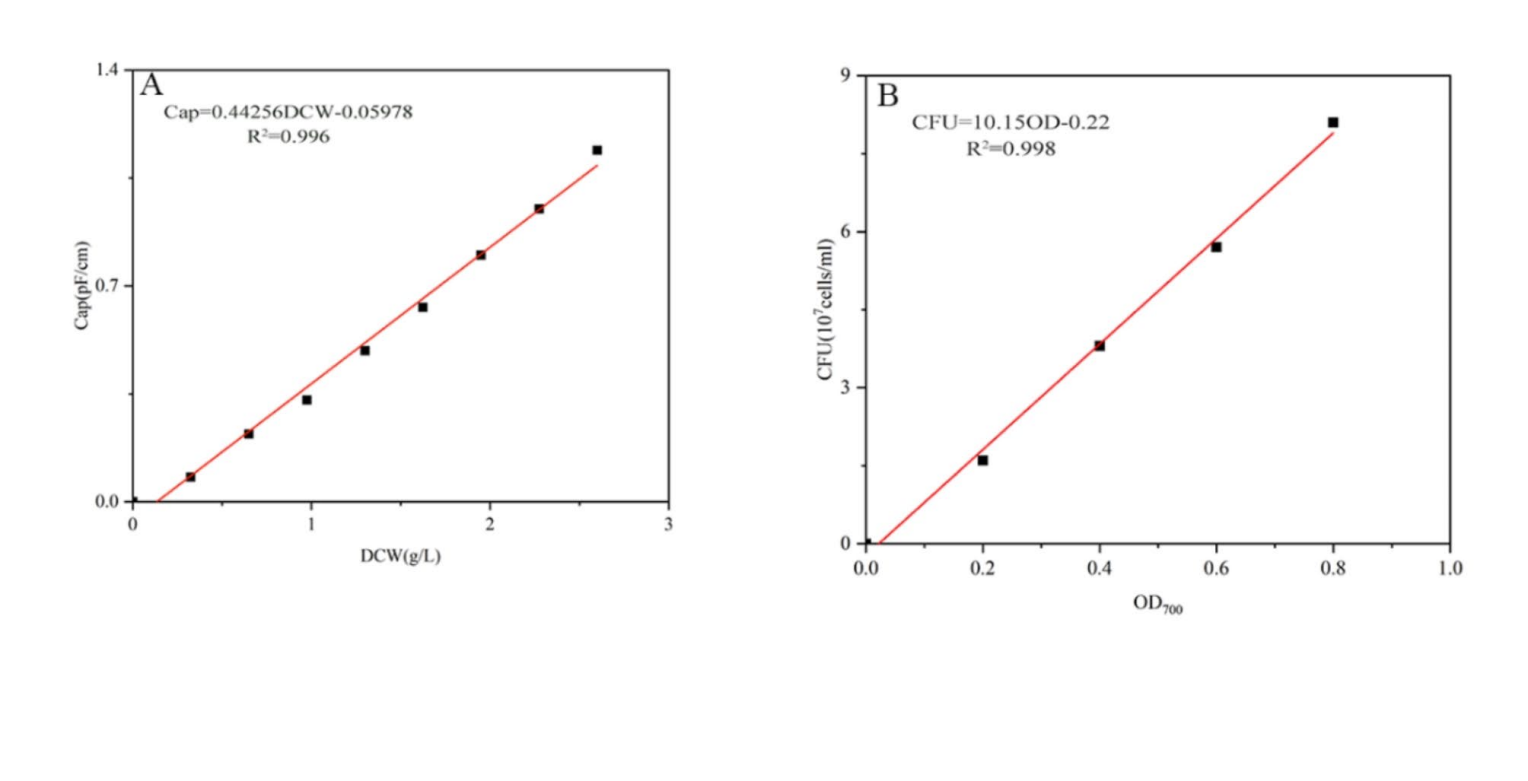
The model for detecting viable *R. sphaeroides* cells based on DS: (**A**)The relationship between capacitance (Cap) and the dry cell weight (DCW). (**B**) The relationship between OD_700_ and CFU

Capacitance can be measured by DS at different frequencies, with the suitable frequency being influenced by cell morphology, size, and type (Kiviharju et al. 2008). Bacteria suitable measurement frequency is usually at 680 kHz (Z.-J. Wang, Shi, et al., 2016). To identify whether the capacitance measured at 680 kHz can accurately detect and reflect the biomass of *R. sphaeroides*. The relationship between capacitance and viable cell concentration was first studied using *R. sphaeroides* reference materials, which contained a known quantity of viable cells. The results (Fig. 1A) revealed an excellent linear relationship between capacitance and the number of viable cells, with R^2^ = 0.996, demonstrating that the DS can accurately detect the viable cells number of *R. sphaeroides* at 680 kHz. Moreover, we modeled the relationship between OD and CFU using the *R. sphaeroides* reference materials. Since the number of viable cells in the reference materials was known, this allowed to accurately model the relationship between OD and CFU, avoiding the error associated with using fermentation broth for CFU detection. The model (Eq. 1) between OD and CFU showed an excellent linear relationship as depicted in Fig. 1B (R^2^ = 0.998). Therefore, DS can be used to measure the biomass of *R. sphaeroides* offline at 680 kHz. However, for online measurement, capacitance may be affected by agitation, aeration, morphology, and culture broth (Kiviharju et al. 2008).

1 $$\:\text{CFU=10.15OD}\text{-0.22\:\:}$$

### The relationship of on-line capacitance and off-line biomass measurements

Previous researches have reported a good linear correlation between online capacitance measured by DS and OD or DCW (Horta et al. 2015). However, to our knowledge, there are few studies focused on real-time biomass monitoring in CoQ_10_ fermentation using capacitance probes. Since CoQ_10_ fermentation by *R. sphaeroides* is characterized by a complex medium, high agitation speed, changes in cell morphology, and high cell density fermentation, which may affect online capacitance measurement. Therefore, the response relationship between the OD_700_ and capacitance of *R. sphaeroides* during the fermentation process was first studied.

**Fig. 2:**
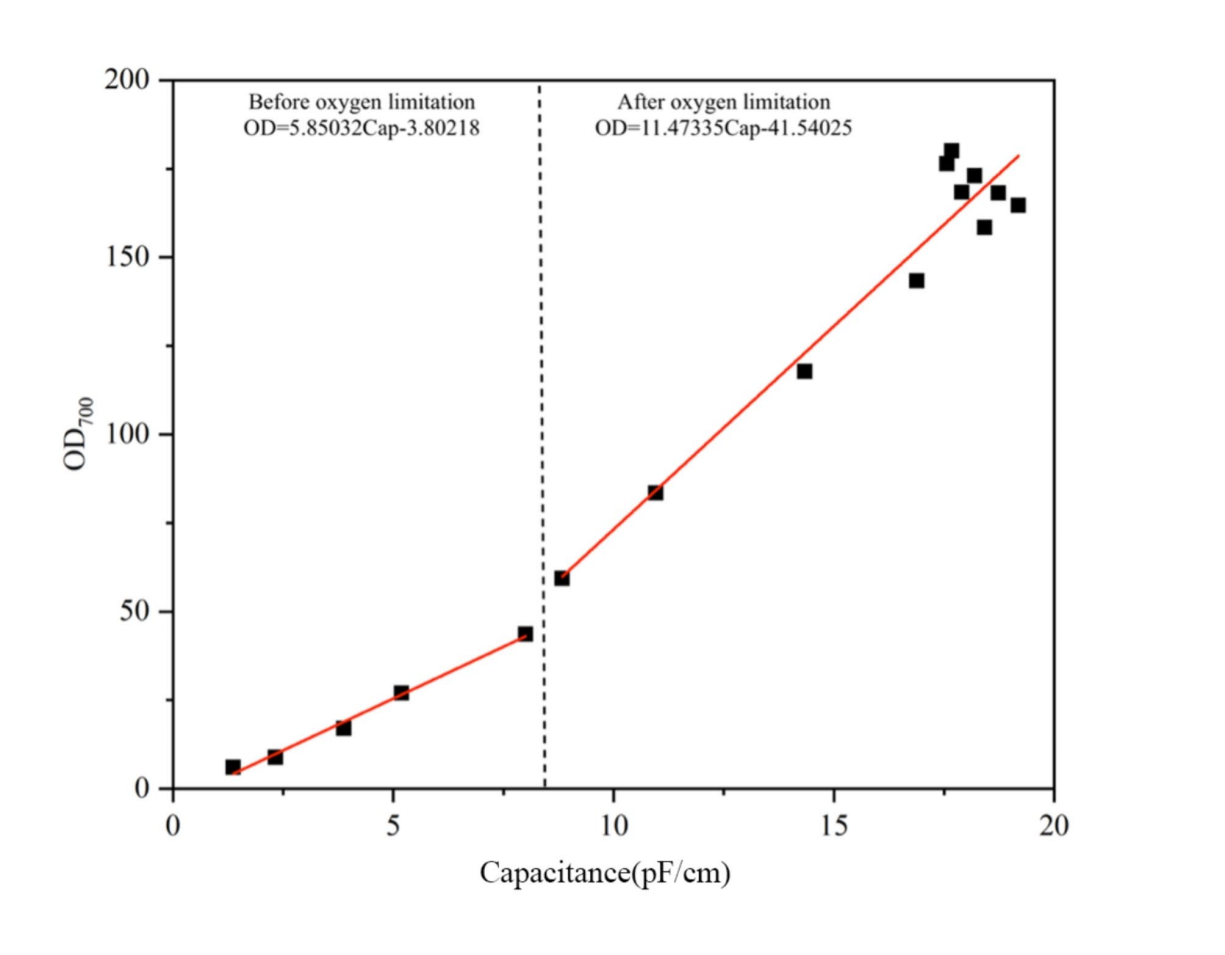
The relationship between Capacitance and OD_700_ during the fermentation process

Figure 2 shows the model of online capacitance and OD_700_ during CoQ_10_ fermentation, established separately for periods before and after oxygen limitation. The results show that before the oxygen limitation, the linear relationship between capacitance and OD_700_ was significant, with a correlation value of R^2^ = 0.99 (Eq. 2). After entering the oxygen-limited phase, the R^2^ decreased to 0.931 (Eq. 3), which may be related to change in the morphology and death of *R. sphaeroides* cells, which influenced the capacitance and OD_700_ measurements. Despite this, the linear relationship between OD_700_ and capacitance remained significant. A similar divergent trend between DCW was observed during the VB_12_ fermentation process after the morphology of *P. denitrificans* changed (Z.-J. Wang, Shi, et al., 2016). Therefore, the online capacitance and OD_700_ have a good linear correlation in the *R. sphaeroides*, allowing for the rapid, accurate, and straightforward determination of viable cells during the fermentation process using DS. Moreover, the model between OD and capacitance was established.

2 $$X=-3.8022+5.8503\times \text{Cap}$$

3 $$X=-41.51+11.473\times \text{Cap}$$

Accurately and rapidly detecting the quantity of viable cells is crucial for process control and optimization. However, using CFU to detect viable cells presents several challenges, including data lag, bacterial contamination risks, and low accuracy. These issues may lead to missing critical control and optimization points in the fermentation process, affecting the final yield. As an online sensor, DS is able to detect viable cells in real time, enabling timely regulation of the fermentation process. Research has shown that erythromycin production increased by 4% when propanol feed was regulated using online capacitance monitoring (Guo et al. 2016). Furthermore, by combining online capacitance data with physiological parameters, we can better understand the physiological state and key metabolic parameters of *R. sphaeroides*, which could be highly beneficial.

### The physiological metabolism of CoQ_10_ fermentation of *R. sphaeroides*

DS enables the online detection of viable *R. sphaeroides* cells. In addition, nutrient is a critical factor influencing the number of viable cells and biosynthesis, especially using the complex media for biosynthesizing of secondary metabolites (Reshi et al. 2023). DS can also monitor conductivity, which provides insight into nutrient consumption within the culture system (Z.-J. Wang et al. 2016b). In addition, OUR has a significant influence on CoQ_10_ yield (Z.-J. Wang et al. 2020). To comprehensively understand the physiological responses to physical conditions and identify process-critical factors, it is crucial to analyze the interactions and mutual influences between various physical and physiological parameters in detail (Wang et al. 2009).

**Fig. 3:**
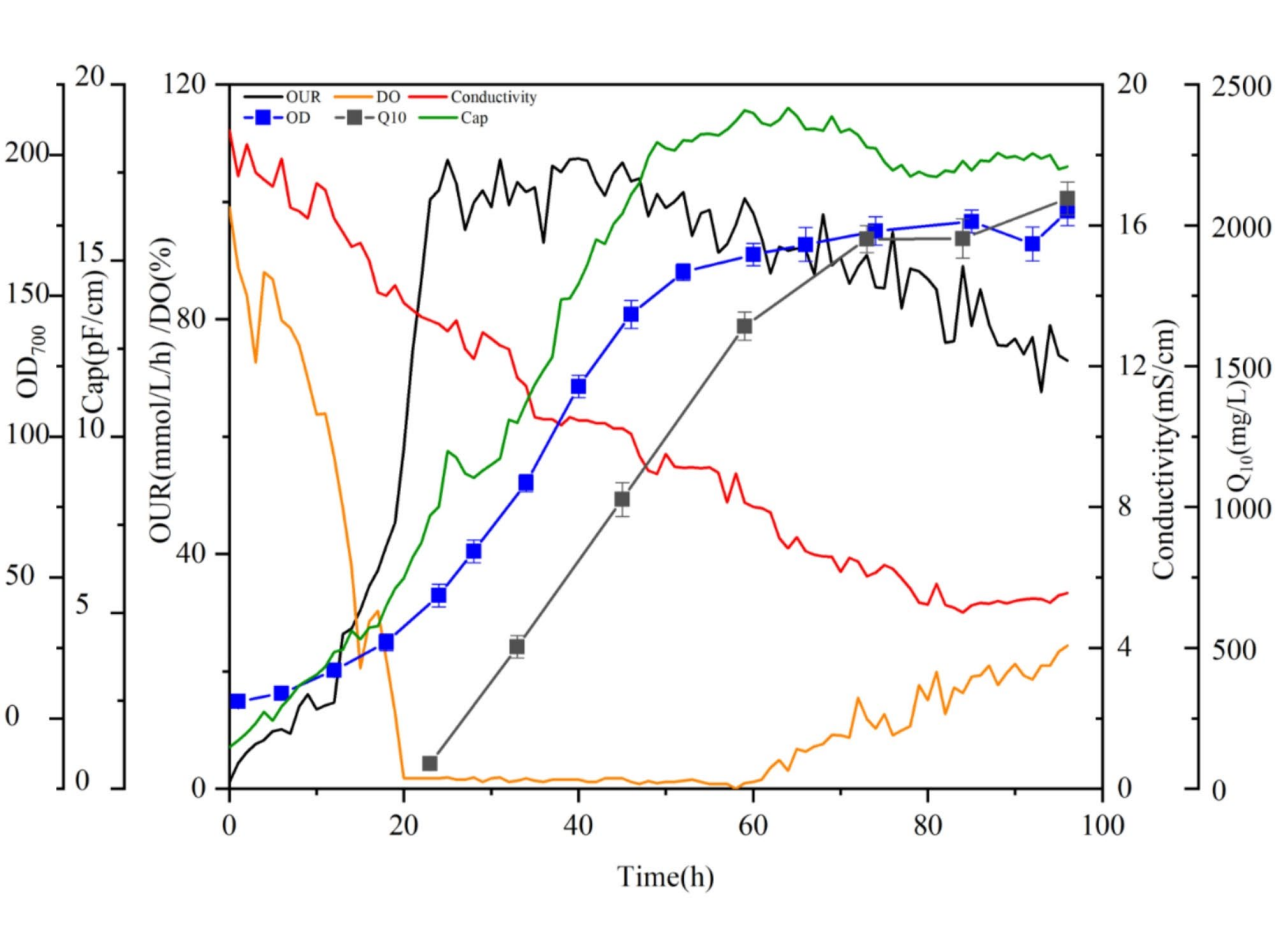
Profiles of physiological parameters in CoQ_10_ (CoQ_10_) fermentation process

Figure 3 shows the profiles of capacitance, conductivity, OD_700_, DO and CoQ_10_ concentration during the fermentation process. Before 20 h, DO decreased with the growth of *R. sphaeroides*, and the capacitance increased with OUR elevated. At the same time, nutrient consumption caused a decline in conductivity. After 20 h, CoQ_10_ biosynthesis commenced under oxygen restrictive conditions, with OUR stabilized at 105 ± 5mmol/L/h. With cell growth and CoQ_10_ biosynthesis continued, nutrient depletion further reduced conductivity, which dropped from 18.3 to 9 ± 0.5 pF/ms after 50 h. Meanwhile, DO stared to rise, capacitance and OUR began to decrease, and CoQ_10_ biosynthesis rate appeared to greatly decrease. This may be attributed to nutrient exhausting in the fermentation broth, which reduces the cell viability and leads to the decay of *R. sphaeroides*, ultimately inhibiting OUR.

Therefore, due to the lack of sufficient nutrients after 50 h, the decreased viability of *R. sphaeroides* was accompanied by a decline in the number of viable cells, which leads to a decrease in capacitance. With the decline of viability and viable cells, OUR also reduced, which further led to the decline in the CoQ_10_ biosynthesis rate. Since CoQ_10_ is an essential electron carrier in the respiratory chain, respiration intensity affects yield (Cluis et al. 2007), which may be influenced by cell viability and number of viable cells. Recent research reported that modifying the redox respiratory chain in *R. sphaeroides* improved CoQ_10_ yield by 7.6% (Zhang et al. 2018). Moreover, OUR influences the cell morphology, which in turn influences yield (Papagianni and Mattey 2006). Through optimizing the agitation to regulate the morphology of *Aspergillus oryzae*, Rhizomucor miehei lipase activity increased by 29.1% (Li et al. 2022). *R. sphaeroides* morphology also changed during the fermentation process, Wang et al. found that high cell volume size was achieved using the OUR-Stat strategy, which improved the CoQ_10_ yield by 15.4% in *R. sphaeroides* (Z.-J. Wang et al. 2020). Therefore, controlling the oxygen supply level is a useful and challenging strategy.

### Cells’ morphology and CoQ10 biosynthesis

**Fig. 4:**
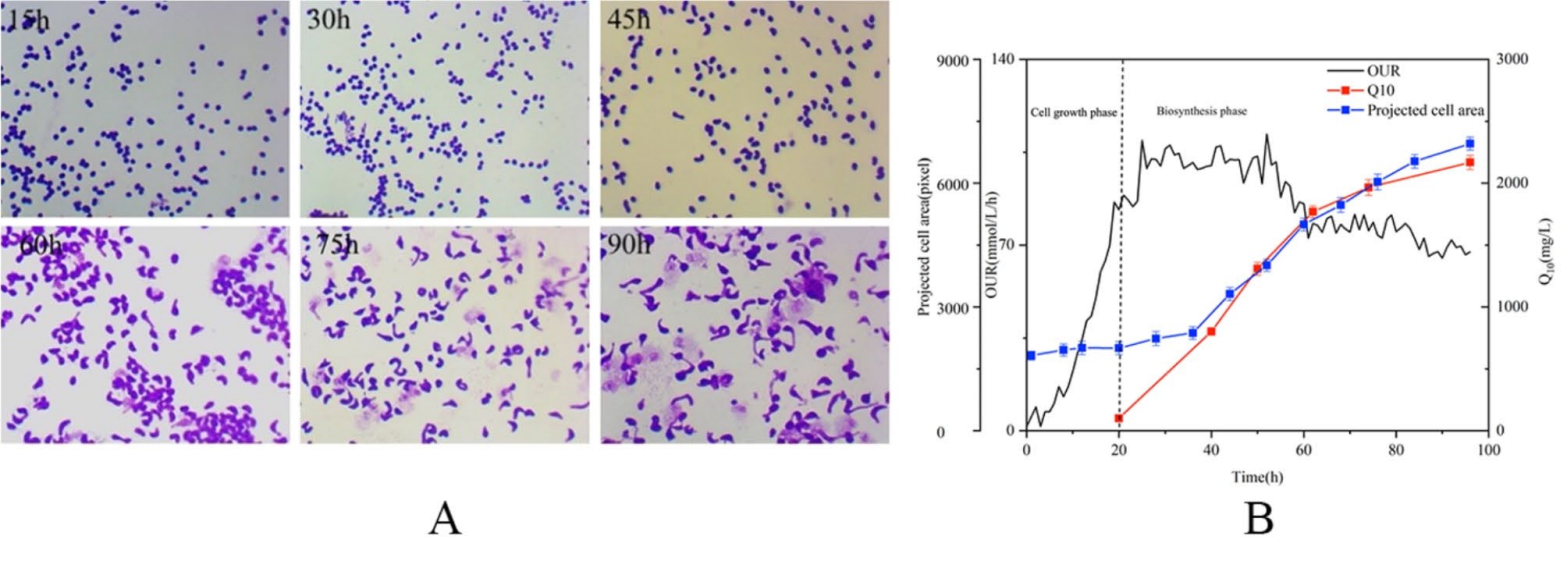
The relationship between cell morphology and CoQ_10_ biosynthesis: (**A**) The morphological differentiation of *R. sphaeroides* during the CoQ_10_ fermentation process. (**B**) The relationship among the average projected area of cells, CoQ_10_ production, and OUR during the fermentation process

Morphology changes are commonly observed during the fermentation process, and it is a signal for cell shift from growth to biosynthesis (Sizova et al. 2011). In addition, the morphology of bacteria is affected by oxygen, especially the fermentation processes exist oxygen limitation (Papagianni and Mattey 2006). Notably, the evident morphology changes of *R. sphaeroides* during the fermentation process were also observed (Fig. 4A). However, there are hardly any reports on the relationship between cell morphology, OUR, and product biosynthesis in *R. sphaeroides*.

Through observing *R. sphaeroides* morphology (Fig. 4A), it is evident that the CoQ_10_ fermentation process was divided into two distinct stages: the increase in the cell number and the subsequent expansion of the individual cells. We further established the relationship among the average projected area of the cells, CoQ_10_ biosynthesis, and OUR during the fermentation process (Fig. 4B). Before 20 h, *R. sphaeroides* was in a phase of numerical growth, accompanied with OUR increase. There was no change in cell morphology, and CoQ_10_ did not accumulate. After 20 h, OUR gradually reached a stable state, and CoQ_10_ began to accumulate. At the same time, the cells morphology stared to expand, and the projected area gradually increased. After 60 h, with the decrease of OUR, the biosynthesis rate of CoQ_10_ and the increase in cell projected area both decreased, indicating that the decrease of OUR has a conducive relationship with the cell expansion and product biosynthesis. At the end of 96 h, the cells projected area (6958.4 8(pixel) increased 3.8 times compared to that of the normal morphology (1821.8(pixel). Hence, OUR significantly impacted the change of *R. sphaeroides* morphology. As the morphology of *R. sphaeroides* expanded after oxygen limitation, CoQ_10_ was rapidly biosynthesized and accumulated. Moreover, it is commonly reported that there is a direct relationship between cell morphology and the secondary metabolite yield in industrial fermentations. Since CoQ_10_ is mostly distributed in the membrane, optimal cell morphology is crucial for improving production. Wang et al. have found that optical cell morphology and highest yield were obtained under proper OUR conditions (Z.-J. Wang et al. 2020). However, this study mainly focused on the oxygen uptake rate with per unit volume unit and ignored the Q_O2_, and its effect on morphology.

### Q_O2_, *R. sphaeroides* morphology, and CoQ_10_ production

Q_O2_ can more accurately reflect the cell oxygen supply situation. Utilizing the online detection of capacitance by DS provides an efficient and feasible strategy to control Q_O2_ precisely. Q_O2_ can be calculated using Eq. 4, where X represents the viable cells number (Deshpande & Heinzle, 2004). By combining the relationship between OD and CFU (Eq. 1), which was established using the reference materials of *R. sphaeroides*, and the relationship between OD and capacitance during the fermentation process (Eq. 2 and Eq. 3), the model between CFU and Cap was established before and after oxygen limitation (Eq. 5, Eq. 6).

4 $$\:\text{OUR\:=X\:Q}\text{O}\text{2}\text{\:\:\:\:\:\:\:}$$

5 $$\:\text{X(CFU)\:=59.38Cap-38.81\:\:\:\:\:\:\:}$$

6 $$\:\text{X(CFU)\:=116.45Cap-421.63\:\:\:\:\:}$$

**Fig. 5:**
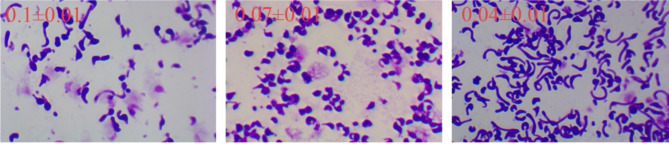
Cell morphology at different Q_O2_ levels during fermentation for 80 h

Through the model, Q_O2_ were controlled at 0.03–0.05 × 10^− 7^mmol/cell/h, 0.06–0.08 × 10^− 7^mmol/cell/h, 0.09–0.11 × 10^− 7^mmol/cell/h. Physiological parameter results (Table 1) showed that the Q_O2_ controlled at 0.06–0.08 × 10^− 7^mmol/cell/h was most conducive to CoQ_10_ biosynthesis, with the final yield reaching 2552 mg/L. The yield was 17.2% and 28.3% higher compared to the Q_O2_ level of 0.09–0.11 × 10^− 7^mmol/cell/h and 0.03–0.05 × 10^− 7^mmol/cell/, respectively. Moreover, the production rate and specific production rate were obviously higher at this optimal Q_O2_ level. In addition, Fig. 5 shows that cell morphology also significantly different at different Q_O2_ levels after 80 h of fermentation. At the lowest Q_O2_ level of 0.03-0.05mmol/cell/h×10^− 7^, cell morphology clearly stretched, and the biomass was the lowest (Table 1), likely due to serious oxygen supply limitation. At the highest Q_O2_ level of 0.09–0.11 × 10^− 7^mmol/cell/h, there existed cell morphological differences. The optimal cell morphology was achieved at the Q_O2_ level of 0.06–0.08 × 10^− 7^mmol/cell/h, suggesting that Q_O2_ has a significant influence on the cell morphology, which in turn affects production. Yoshida et al. also found noticeable differences in the thickness of the cell membrane under different oxygen conditions. The thickness of the cell membrane was 0.4 μm under low oxygen conditions, while under high oxygen conditions, the thickness of the intracytoplasmic membrane was only 0.2 μm. The thickness of the cell membrane increased with lower oxygen supply and decreased with higher oxygen supply, which affected CoQ_10_ biosynthesis (Yoshida et al. 1998). Since the cell membrane is the main site for CoQ_10_ biosynthesis, a thicker cell membrane provides more space for CoQ_10_ biosynthesis and storage. In addition, the Q_O2_ controlling strategy was also applied in other fermentation processes. For instance, controlling the Q_O2_ levels in VB_12_ biosynthesis optimized cell morphology and improved production by 14.7% (Z.-J. Wang et al. 2016b). Similarly, in polyhydroxyalkanoates (PHAs) fermentation, Q_O2_ controlling strategy also has been applied for optimizing the fermentation process (Li et al. 2014). Therefore, by detecting capacitance through DS, real-time viable cell number and Q_O2_ situation could be simultaneously obtained and controlled during fermentation, providing a reliable strategy to enhance CoQ_10_ production.

**Table 1:** The influence of Q_O2_ control level (20–96 h) on the synthesis of CoQ_10_

Factors	Q_O2_ control strategies		
Q_O2_ (10^− 7^mmol/cell/h)	0.1 ± 0.01	0.07 ± 0.01	0.04 ± 0.01
CoQ_10_(mg/L)	2178	2552	1989
Production rate(mg/L/h)	22.7	26.5	20.7
Biomass weight(g/L)	92.6	87.1	80.4
Specific production rate(µg/g/h)	245.1	304.2	257.5
Glucose consumption rate(g/L/h)	4.38	2.89	1.52
Specific glucose consumption rate(mg/g/h)	47.4	33.2	18.9

### Optimization of nutrients concentration directed by DS

Q_O2_ levels significantly affect cell morphology, thereby affecting the production of CoQ_10_. However, CoQ_10_ production was also influenced by the vitality and number of viable cells of *R. sphaeroides*, which decreased during the later stage of fermentation. This decline may be related to nutrient deficiency. Therefore, the online capacitance and conductivity were monitored to guide the nutrient addition to alleviate this effect. By controlling the timing and rate of nutrient addition, conductivity was maintained at 9.0 ± 0.5 mS/cm (A), 7 ± 0.5 pF/cm (B), and left uncontrolled (C).

**Fig. 6:**
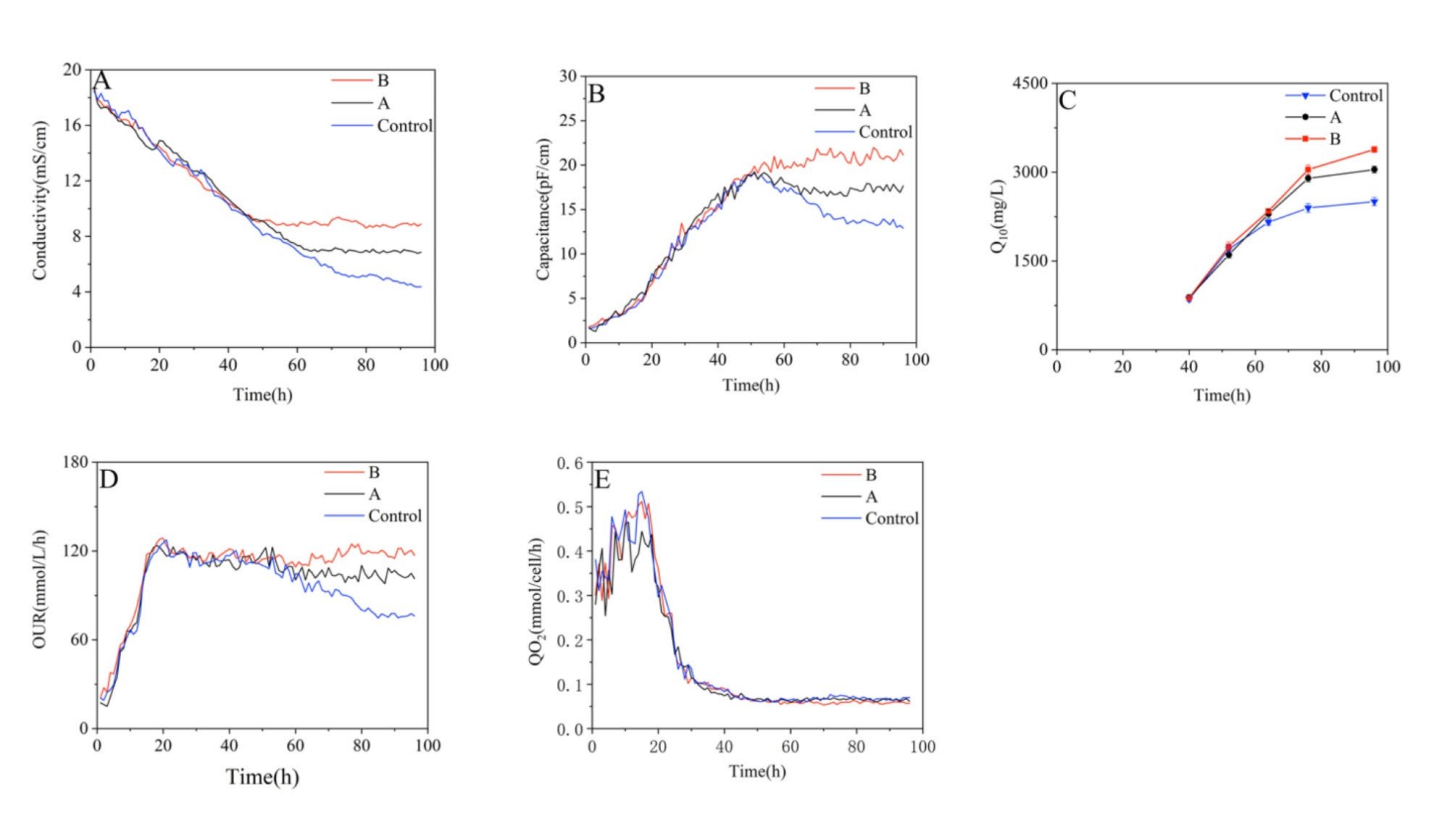
Profiles of physiological parameters under different nutrient feeding strategy during the CoQ_10_ fermentation. (**A**) Conductivity; (**B**) Capacitance; (**C**) CoQ_10_ titer; (**D**) OUR; (**E**) Q_O2_

In group A, OUR maintained at a relatively stable level (Fig. 6D), demonstrating that the addition of nutrients maintains cell viability and viable cell numbers. From the capacitance date (4.6B), it can also be observed that there was no decrease after nutrients added at 50 h, maintaining a constant level of 20 ± 1 mS/cm until the end of fermentation. In group B, a decrease in capacitance was evident after 50 h, with capacitance reducing to 18 ± 0.1 mS/cm by 60 h. After added nutrient, the capacitance remained stable at 18 ± 1 mS/cm until the end of fermentation. Group C exhibited a continuous decrease in capacitance, dropping to 12.91 ± 0.1 mS/cm in the late stages of fermentation. The profiles of Q_O2_ (Fig. 6E) showed that Q_O2_ was maintained at the highest level before the oxygen limitation, but with rapid cell growth, Q_O2_ dropped to 0.08 × 10^− 7^mmol/cell/h after 40 h. The maximum CoQ_10_ production (3384 mg/L) was obtained under the controlled conductivity conditions of 9.0 ± 0.5 mS/cm (Fig. 6C), which was 11.1% and 35% higher than that of group A and control, respectively. Since CoQ_10_ is mainly presented in the cell membrane, maintaining a high concentration of viable cells and achieving optimal cell morphology are crucial factors affecting CoQ_10_ biosynthesis in the late fermentation stage. The use of DS to add nutrients reduces cell decay and help maintains the vitality of the cell. In addition, controlling appropriate Q_O2_ level through online capacitance monitoring ensured the respiratory metabolism intensity, which in turn leads to optimal cell morphology. The research has reported that through optimizing the broth conductivity using DS, the cell growth rate and taxol productivity can be enhanced in Taxus (Z.-J. Wang et al. 2016b). The real-time detection of the conductivity and capacitance by DS could also be applied to increase CoQ_10_ yield, which is an effective strategy for large-scale CoQ_10_ production.

## Conclusion

Morphology and viable cell are two key factors that significantly impact CoQ_10_ biosynthesis, both of which are closely related to oxygen supply. In this study, we built a model for detecting viable cells online through DS during the fermentation process, combined with the online parameter OUR, Q_O2_ was accurately controlled online. By controlling the Q_O2_ level at 0.07 ± 0.01 × 10^− 7^mmol/cell/h, the higher CoQ_10_ biosynthesis and optimal cell morphology ware achieved, which got a 28.3% increase in CoQ_10_ production than that controlled at 0.04 ± 0.01 × 10^− 7^mmol/cell/h. Meanwhile, based on the multi-parameters analysis, an online precise nutrient feeding strategy directed by the conductivity control was successfully established. This strategy effectively addressed the decline and decay of *R.sphaeroides* in the later fermentation stage, resulting in the highest CoQ_10_ production of 3384 mg/L, 35% higher than that of the control. This approach represents an effective strategy for large-scale CoQ_10_ production.

## Declarations

### Availability of date and materials

The data supporting the conclusions are included in the main manuscript.
